# Using a case study approach to document ‘preferred practices’ in mass drug administration for trachoma

**Published:** 2014

**Authors:** Esmael Habtamu, Anne Heggen, Danny Haddad, Paul Courtright

**Affiliations:** Trachoma Research Programme Officer, The Carter Center, Bahirdar, Ethiopia.; Public Health Consultant, Kampala, Uganda.; Director Global Ophthalmology, Emory Eye Center, Emory University, Atlanta, GA, USA.; Director, Kilimanjaro Centre for Community Ophthalmology, Cape Town, South Africa.

**Esmael Habtamu**

**Anne Heggen**

**Danny Haddad**

**Paul Courtright**

Worldwide, trachoma has blinded about 1.2 million people and resulted in visual impairment in a further 1 million people. It is estimated that about 230 million people live in trachoma-endemic areas.^1^

Trachoma elimination programmes use the SAFE strategy: Surgery to correct trichiasis, Antibiotic distribution to treat and prevent chlamydial infection, and Facial cleanliness and Environmental improvements to reduce transmission within communities. Treating all individuals within households and communities with antibiotics reduces the pool of trachoma infection in the population and controls transmission.^2^,^3^ WHO guidelines indicate mass drug administration (MDA) to entire districts where the prevalence of active trachoma in children is more than 10%.^4^ The most commonly used antibiotics are either a single dose oral azithromycin (Zithromax®) (20mg/kg) or 1% tetracycline eye ointment applied twice daily for six weeks.^5^,^6^ MDA of Zithromax® has been extensively conducted in trachomaendemic countries following the donation of the antibiotic by Pfizer Inc. through the International Trachoma Initiative (ITI).^3^ During the year 2012, over 48 million people were treated with the antibiotic.

Trachoma control programmes use various approaches to conduct Zithromax® MDA.^3^ The MDA approaches are influenced by the health system of the country and availability of resources. Programmes generally use either community-based volunteer drug distributers or front-line health workers to distribute the antibiotic. Some programmes implement a campaign approach while others implement a staggered approach. In recent years, external pressure for integrated MDA with other neglected tropical diseases (NTDs) has greatly influenced Zithromax® MDA strategies.^3^

Nevertheless, there has been no evidence of the effectiveness and efficiency of the various approaches and strategies for Zithromax® MDA. ‘Preferred practices’ of community-wide Zithromax® MDA have not been adequately documented or shared among programmes. In order to reach the goal of global elimination of blinding trachoma by 2020, there will be a need to more than double the current distribution efforts.^7^ However, many programmes are struggling to determine the best approaches to efficiently and effectively implement Zithromax® MDA. It was, therefore, crucial to document preferred or best practices of Zithromax® MDA to guide new and existing trachoma control programmes.

## What is a preferred practice?

A preferred practice is generally defined as a ‘technique or methodology that, through experience and research, has proven reliable to lead to a desired result.’^8^ In respect to trachoma MDA it can be used to refer to ‘knowledge about what works in specific situations and contexts to achieve high coverage of MDA’.^8^ Documenting practices that did not work in a particular programme and why they did not work is also a fundamental component of preferred practices.^8^

The World Health Organisation African Regional Office (AFRO) guide to identify and document best practices in health programmes includes 9 characteristics (see panel). Among these, preferred practices should at least be effective (achieve measurable results), efficient (implemented with reasonable resources and time) and relevant (address a priority health problem).^8^

Various approaches can be used to document preferred practices. We implemented a four-part methodology to identify and document Zithromax® MDA preferred practices.

**Preferred practice characteristics**

- effectiveness
- efficiency
- relevance
- ethical soundness
- sustainability
- possibility of duplication
- partnership
- community involvement
- political commitment

1. Conduct a literature review on MDA (any disease or condition).
2. Distribute a standardised form to all national coordinators of countries where Zithromax® MDA is implemented to establish baseline data on the health systems of each country and to learn how Zithromax® MDA is conducted in particular settings.
3. Implement a multi-country case study approach to learn how Zithromax® MDA was conducted in practice.
4. Carry out an expert review of the case studies to identify preferred practices generated from the cases studies and lessons learned (which led to the drafting of a preferred practices manual).

Here we will review how the case study approach was developed and implemented to help eye care programme personnel and researchers consider how a case study I approach may be useful for addressing other eye care I interventions.

**Figure:**
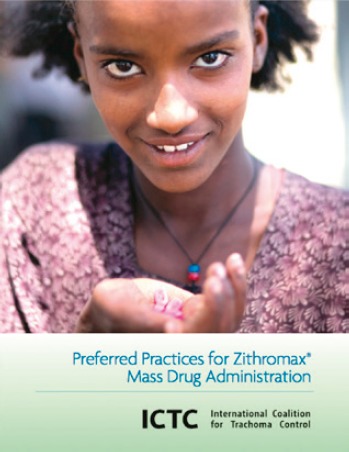
The preferred practices manual

## What is a case study approach?

A case study approach to I research is particularly useful to understand and explore the practical changes, variations and processes of a complex and multifaceted intervention or programme in a real-world context.^9^,^10^ Unlike other experimental designs, case studies are useful in presenting occurrences and processes in their natural context.^10^ According to Crowe and colleagues, a case study helps to “capture information in more explanatory ‘how’, ‘what’ and ‘why’ questions, such as ‘how is the intervention being implemented and received on the ground?’ and ‘what gaps exist in its delivery?’ or ‘why one implementation strategy might be chosen over another?’”.^9^ Among the various types of case study designs, we have chosen to implement the “collective” case study approach where we studied and documented the various components and processes of Zithromax® MDA in a number of different settings.^9^

There were six steps we undertook in conducting and using the case studies.

## Step 1 Definition and selection of ‘cases’

The ‘cases’ in our study were MDA programmes. The criteria for selection included the following.

- The MDA programme should have been in place for at least 2 years and the programme should have made some changes (matured) based upon the experiences of the previous year or two.
- The MDA programme should have a reasonable MDA administrative coverage (ideally 80% or more of the total population in a targeted district receiving treatment) although, as noted above, much also can be learned from programmes that are not achieving their targets.
- A variety of different approaches (centralised versus decentralised planning, community volunteers versus health workers as distributors, vertical trachoma MDA versus integrated NTD MDA) to obtain a range of experiences. Seven case studies were conducted on seven trachoma control programmes from four countries (Ethiopia, Kenya, Nigeria, and Uganda).

## Step 2 Creation of a conceptual framework

Simply put: what information do we need to collect, why do we need this information, and how are we going to collect this information? The framework was developed based on the literature review and through input from a working group of experts. The conceptual framework was the common reference and guide for data collection in all settings.

At national and regional level, information was collected on:

- planning and coordination
- financing
- integration with other NTDs or services
- reporting.

At the level of MDA implementation (usually district level), information was collected on:

- pre-MDA advocacy
- personnel training
- delivery of Zithromax® to distribution areas
- experiences of integrating Zithromax® MDA with MDA for other NTDs
- micro-planning
- recording of treatment and adverse events
- participant incentives
- supervision
- timing of MDA
- perceptions of the recipients of Zithromax® MDA.

The framework was not designed to be restrictive; it was possible to expand areas of exploration, as needed.

**Figure:**
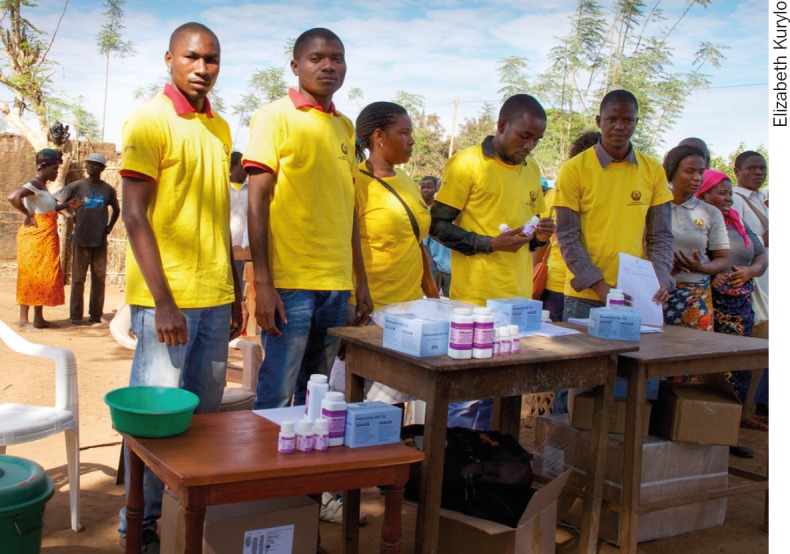
Mass drug administration for trachoma. MOZAMBIQUE

## Step 3 Implementation of the case studies

The case study approach requires collecting quantitative and qualitative data from multiple sources. Qualitative methods such as interviews, focus group discussions and observations are particularly useful and are commonly implemented.^9^ These methods, in addition to a review of records, were used for these case studies. The first case study was carried out jointly by two of the authors in order to test out the framework, get a sense as to the right balance of interview and observation, decide on the level of detail to include in the final version of the case study, and to jointly highlight characteristics of MDA (from the triangulation of data) that embodied the concepts of effectiveness and efficiency. The remainder of the case studies were conducted in 2011 and 2012.

## Step 4 Analysis and interpretation of data

First, each case study report was reviewed by the planners and implementers of the programmes from which the case study was taken; this was to confirm and clarify all aspects of the case study. Then, the relevant characteristics from each case study were compiled into a coherent grouping (referred to as preferred practices) which were sent to the expert group for input.

## Step 5 A meeting of experts and implementers to review the preferred practices

A one-day meeting in Ethiopia focused on reviewing the preferred practices in the Ethiopian context. This information, plus the full body of preferred practices, were then presented at an experts' meeting in Cape Town, South Africa. These meetings enabled the team to get valuable input as well as to obtain agreement on the best way to use the information.

## Step 6 Establishment of a small writing group with participation from a wide group of stakeholders

Individuals on the writing group were requested to prepare specific sections of the preferred practices manual, which then underwent compilation and editing. The result can be found online at www.trachomacoalition.org.

The entire process took almost two years to complete and it should not be assumed that a preferred practice manual is, by itself, sufficient to improve the effectiveness and efficiency of MDA for Zithromax®. Based upon the work, however, a number of capacity building activities have been identified and will be undertaken over the coming few years. Improvements in effectiveness and efficiency are the primary goals.

The International Coalition for Trachoma Control (ICTC) has adopted the preferred practices as basis for its support to national programmes that are funded through ICTC consortia. During the start-up workshops for the Queen Elizabeth Diamond Jubilee Trust's Trachoma Initiative, preferred practices and the adoption of these to local settings have become the standard. In order to ensure that the goal of elimination of blinding trachoma will be reached, there is an urgent need to ensure that high quality programmes are implemented from the start and are reaching as many people as possible.

The Kilimanjaro Centre for Community Ophthalmology (KCCO) was the lead partner on behalf of the International Coalition for Trachoma Control (ICTC), with support from ITI, to carry out this project.
